# The Histone Demethylase Jarid1b (Kdm5b) Is a Novel Component of the Rb Pathway and Associates with E2f-Target Genes in MEFs during Senescence

**DOI:** 10.1371/journal.pone.0025235

**Published:** 2011-09-27

**Authors:** Jeroen H. Nijwening, Ernst-Jan Geutjes, Rene Bernards, Roderick L. Beijersbergen

**Affiliations:** Division of Molecular Carcinogenesis, The Netherlands Cancer Institute, Amsterdam, The Netherlands; Texas A & M University, United States of America

## Abstract

Senescence is a robust cell cycle arrest controlled by the p53 and Rb pathways that acts as an important barrier to tumorigenesis. Senescence is associated with profound alterations in gene expression, including stable suppression of E2f-target genes by heterochromatin formation. Some of these changes in chromatin composition are orchestrated by Rb. In complex with E2f, Rb recruits chromatin modifying enzymes to E2f target genes, leading to their transcriptional repression. To identify novel chromatin remodeling enzymes that specifically function in the Rb pathway, we used a functional genetic screening model for bypass of senescence in murine cells. We identified the H3K4-demethylase Jarid1b as novel component of the Rb pathway in this screening model. We find that depletion of *Jarid1b* phenocopies knockdown of *Rb1* and that Jarid1b associates with E2f-target genes during cellular senescence. These results suggest a role for Jarid1b in Rb-mediated repression of cell cycle genes during senescence.

## Introduction

Senescence is a robust cell cycle arrest that can be triggered by various stress signals such as telomere attrition, oncogene activation or DNA damage, which functions to protect cells against malignant transformation [1],[2]. Senescent cells undergo a series of events leading to marked morphological changes, the expression of senescence-associated β-galactosidase (SA-β-gal) and profound changes in gene expression, including activation of the *INK4A-ARF* locus. The *INK4A-ARF* locus is a potent activator of the p53 and RB tumor suppressor networks that enforce an intricate program including the repression of E2F-target genes required for proliferation [3], [4]. Not surprisingly, the p53 and RB proteins are commonly inactivated by viral oncoproteins such as E1A or SV40LT thereby contributing to cellular transformation. In human fibroblasts it has been found that senescence induction is associated with dramatic changes in chromatin organization and several chromatin modifying enzymes have been identified that modulate the senescence response [5]. Both the *INK4A–ARF* locus and genes controlled by RB and E2F are major targets of epigenetic regulation during senescence. The *INK4A-ARF* locus is repressed by concerted action of polycomb group proteins (PcG), which impose trimethylation of histone H3 Lysine 27 (H3K27me3) and histone demethylases JARID1A (KDM5A) and NDY1 (KDMB2B) that remove H3K4me3 and H3K36me3 from this locus respectively [6], [7], [8], [9], [10]. PcG-mediated repression of the *INK4A-ARF* locus is counteracted by JMJD3 which actively removes methylation on H3K27 [11], [12]. In addition, the promoter regions of E2F-target genes become enriched for H3K9me3 and depleted for H3K4me3 during senescence, which is important for gene silencing and correct execution of the senescence response by the RB tumor suppressor network [13]. RB can be regarded as an adaptor protein that recruits several histone modifiers to create a repressive complex to silence E2F-target genes during senescence [5]. For example, RB has been shown to recruit HDAC1, DNMT1, SUV39H1 and the SWI/SNF complex to E2F-target gene promoters [5], [14], [15]. It has been reported that inactivation of *Suv39h1* prevents induction of oncogene-induced senescence, which underscores H3K9 trimethylation as a critical feature of senescence [16]. These observations suggest a role for RB in heterochromatinization of E2F-target genes in senescent cells. Concordantly, RB depletion prevents heterochromatin formation in human diploid fibroblasts [13]. Recently, it has been found that RB has a specific and non-redundant role during senescence in the repression of transcription of E2F-target genes involved in DNA replication [17]. Moreover, an RB mutant unable to associate with chromatin modifying enzymes could not repress DNA replication during oncogene-induced senescence [18]. However, this RB mutant was not compromised in its ability to repress DNA replication during quiescence or differentiation, underscoring the significant role of chromatin modifying enzymes in repression of DNA replication during senescence.

Based on the observations described above and the association of Rb with several different chromatin remodeling enzymes, we argued that Rb may recruit additional chromatin remodeling enzymes that contribute to the suppression of E2f-target genes. The identification of such enzymes is potentially compromised by the notion that inactivation of the RB pathway only is not sufficient to bypass senescence in both murine and human cells [1]. Using a functional genetic screen in murine models in which abrogation of the Rb pathway is sufficient to bypass senescence we discovered that the histone demethylase Jarid1b (Kdm5b) is a critical component of the Rb-E2f pathway. In addition, we found that Jarid1b (Kdm5b) associates with E2f-target genes during senescence, suggesting it may contribute to the repression of E2f-target genes during senescence.

## Results

### A screen for bypass of senescence in MN-tsLT cells identifies Jarid1b

**Figure 1:**
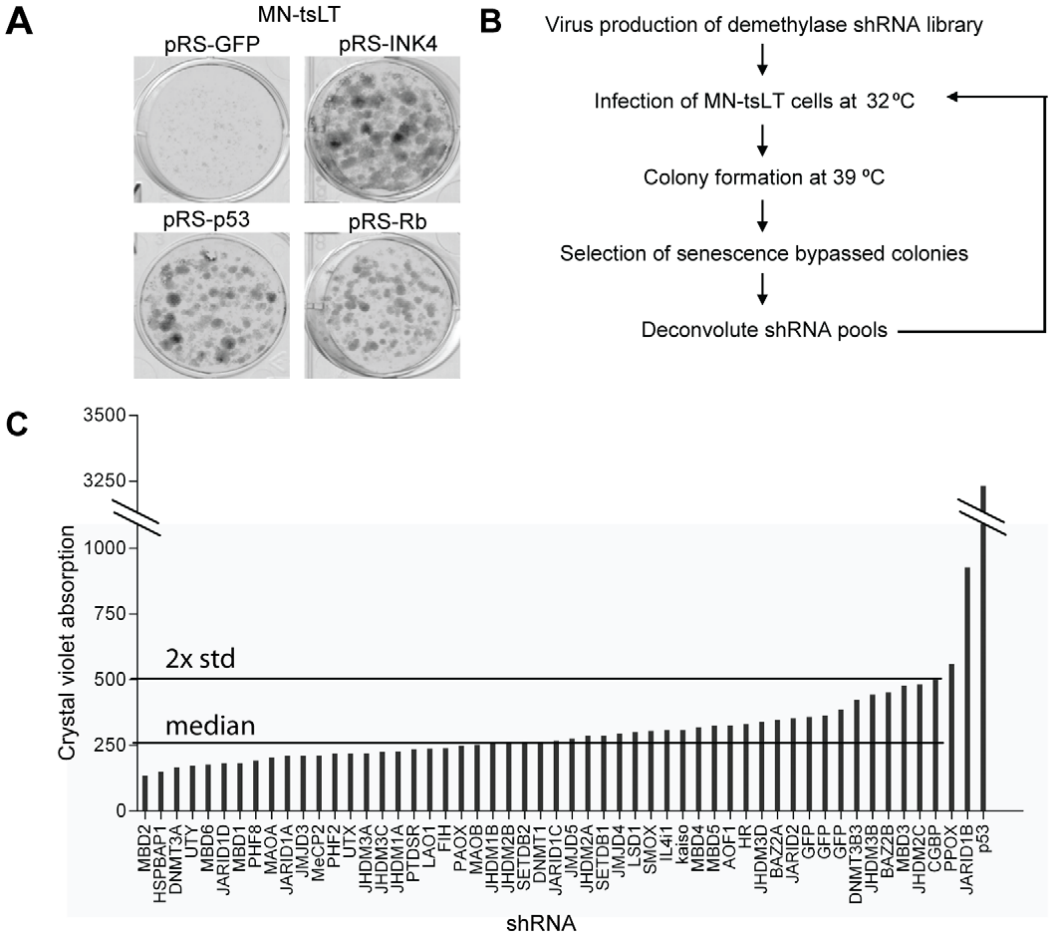
A functional shRNA screen in conditionally immortalized cells. (A) Colony formation assay at 39°C of MN-tsLT cells transduced with pRS-*GFP*, pRS-*Ink4a* (targeting both *Ink4a* and *Arf*), pRS-*p53* and pRS-*Rb* (B) Schematic outline of the senescence bypass screen using MN-tsLT cells. Cells were transduced at the permissive temperature (32°C) with 50 pools of retroviral knockdown vectors targeting candidate chromatin binding and modifying enzymes. Each pool contains 4 unique shRNAs targeting a single transcript. The transduced cells were seeded at the non-permissive temperature (39°C) for a colony formation assay. After 2 weeks cells were fixed and stained with crystal violet. (C) Quantification of the colony formation assay of the shRNA screen in MN-tsLT cells shown are the average absorption and 2× standard deviations (SD) from the median of the samples.

**Figure 2:**
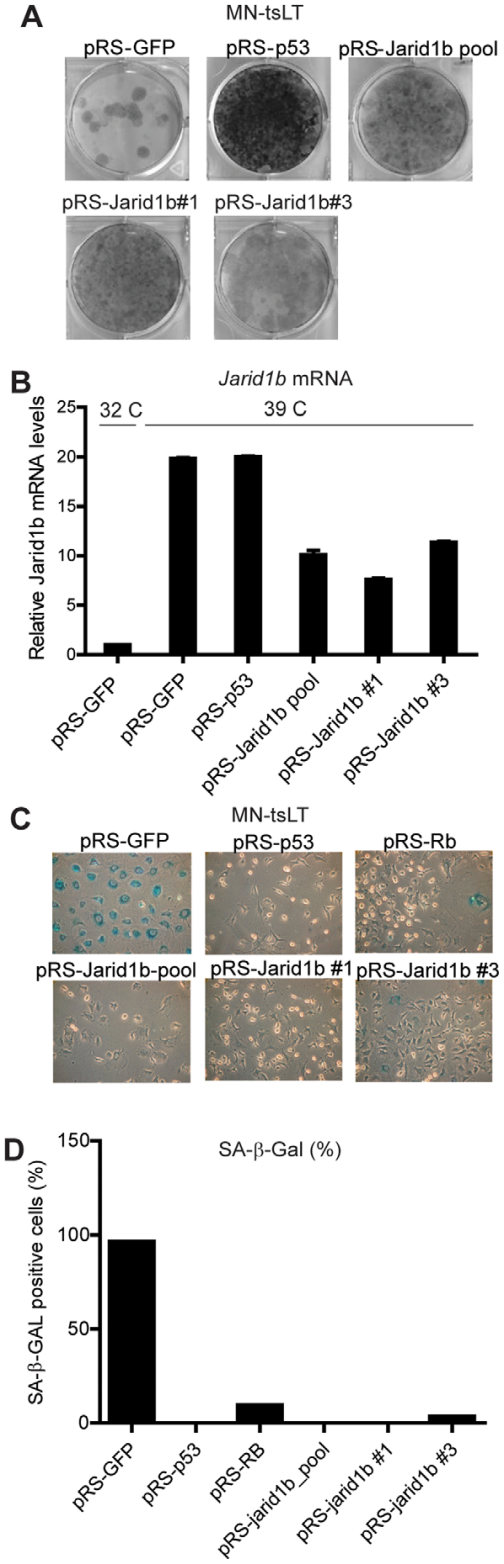
Multiple *Jarid1b*-targeting shRNAs prevent senescence induction of MN-tsLT cells. (A) Colony formation assay of MN-tsLT transduced with two independent *Jarid1b* shRNAs derived from the pool (#1 and #3). pRS-*GFP* was used as a negative control, pRS-*p53* was used as a positive control. (B) Relative *Jarid1b* mRNA expression levels determined by RT-qPCR. Samples are derived from cells shown in (A) and mRNA values were normalized to *Gapdh* and proliferating cells at 32°C. (C) β-galactosidase assay of MN-tsLT cells from (A). (D) Quantification of β-galactosidase positive cells from (C).

To identify novel chromatin remodeling enzymes that specifically cooperate with Rb in tumor suppression, we used a senescence model in which abrogation of the Rb pathway is sufficient to bypass senescence (Figure 1A). The primary mouse striatum cell line MN-tsLT has been conditionally immortalized through the expression of a temperature-sensitive mutant (tsA58) of SV40 large-T antigen (tsLT) [19]. At the permissive temperature MN-tsLT cells proliferate rapidly but they enter into a synchronous senescence-like arrest when shifted to the non-permissive temperature (39°C). MN-tsLT cells arrested at 39°C display several hallmarks of cellular senescence including SA-β-gal positivity, senescent morphology, decreased expression of E2f-target genes and activation of the p53 target gene and cell cycle inhibitor *Cdkn1a* (p21^cip1^) (Figure 2C and D, Supplementary Figure S1B–E). However, similar to murine embryonic fibroblasts (MEFs) and in contrast to human cells [13], senescence-associated heterochromatin foci (SAHF) cannot be detected in MN-tsLT cells. It has been shown previously that inhibition of the p19^ARF^-p53 pathway is sufficient to bypass senescence in this model [20], [21], [22](Figure 1A). We tested whether loss of *Rb1* expression in MN-tsLT cells was sufficient to bypass senescence. As can be seen in Figure 1, the expression of an shRNA targeting *Rb1* (Supplementary Figure S1A) results in the rescue of the senescence phenotype analogous to inactivation of the *Ink4a-Arf* locus or knockdown of *p53*. As such, the dependency on either p53 or Rb in MN-tsLT cells offers an opportunity to find novel components of the p16^INK4A^-Rb pathway.

For this purpose we constructed a retroviral shRNA library consisting of multiple independent shRNAs directed against 50 known and putative chromatin binding and modifying enzymes: Jumonji C (JmjC)-domain-containing proteins, the lysine specific demethylase 1 (LSD1)-like family members, methyl CpG binding proteins and DNA methylases [23], [24]. The shRNAs were pooled in 50 sets of 4 vectors, in which each set of vectors was designed to target a single transcript (Supplementary Table S1).

MN-tsLT cells were transduced at 32°C with the 50 individual sets of shRNAs in a single-well format and seeded for long term clonogenic outgrowth assays (Figure 1B). As a positive control we used a functional shRNA targeting *p53* that was used in previous studies [21], [22]. We used an shRNA targeting green fluorescent protein (*GFP*) as a negative control throughout this study. As expected, knockdown of *p53* prevented senescence induction of MN-tsLT cells (Figure 1A and 1C). Clonogenic outgrowth was quantified by measuring crystal violet absorption. Only wells with an absorption value greater than the median plus 2× standard deviation were considered as hits (Figure 1C). Except for the positive control, only the shRNA pool targeting *Jarid1b* (*Kdm5b*, *Plu-1*, *Rbp2-h1*) [25], [26] fitted these criteria. Jarid1b is a member of the Jarid1 family of H3K4 demethylases [27], [28], [29], [30], [31]. This family encompasses four members (Jarid1a-d) with a high degree of homology [32], all capable of demethylating tri- and di-methylated H3K4 and function as transcriptional repressors. Although shRNA pools against Jarid1 family members a, c and d were present in the library they did not score as hits. On one hand, this could be due to inefficient knock-down of their respective targets but, in contrast to Jarid1b, we did not detect expression of Jarid1a, c or d in MN-tsLT cells (data not shown).

To rule out off target effects [33], each of the individual knockdown vectors of the *Jarid1b* shRNA pool were introduced into MN-tsLT cells and tested for their ability to bypass senescence and their efficiency of knocking down *Jarid1b*. We found two independent shRNAs targeting *Jarid1b* (pRS-*Jarid1b*#1 and #3) that allowed bypass of senescence in MN-tsLT cells. Both shRNAs reduced *Jarid1b* mRNA levels, confirming Jarid1b as an on-target hit (Figures 2A and B). In addition, we found that *Jarid1b* mRNA expression is highly induced when MN-tsLT cells are shifted to the non-permissive temperature, suggesting a role for Jarid1b in the execution of senescence (Figure 2B). Importantly, the expression of *Jarid1b* is not a surrogate marker for the absence of cellular proliferation as MN-tsLT cells that express knockdown vectors against *p53*, and cycling at 39°C, retain high levels of *Jarid1b*.

Next, we analyzed MN-tsLT cells transduced with the two functional *Jarid1b*-knockdown vectors for typical senescence markers [1]. Whereas the negative control vector transduced cells stained highly positive for β-galactosidase, cells expressing the functional *Jarid1b*-knockdown vectors did not or stained weak for β-galactosidase (Figures 2C and 2D). Moreover, *Jarid1b*-knockdown cells did not show a typical senescent morphology observed in the control vector-transduced cells (Supplementary Figure S1B). Expression of two *bona fide* cell cycle markers *Ccna1* and *Pcna* was restored in *Jarid1b* knockdown cells (Supplementary Figure S1C and S1D). Remarkably, levels of *Cdkn1a*, a marker of slowly cycling and senescent cells, remained high in proliferating *Jarid1b*-knockdown cells (Supplementary Figure S1E). Taken together, these data demonstrate that MN-tsLT cells with *Jarid1b* knockdown do not undergo senescence when shifted to the restrictive temperature.

### Jarid1b functions in the Rb pathway

**Figure 3:**
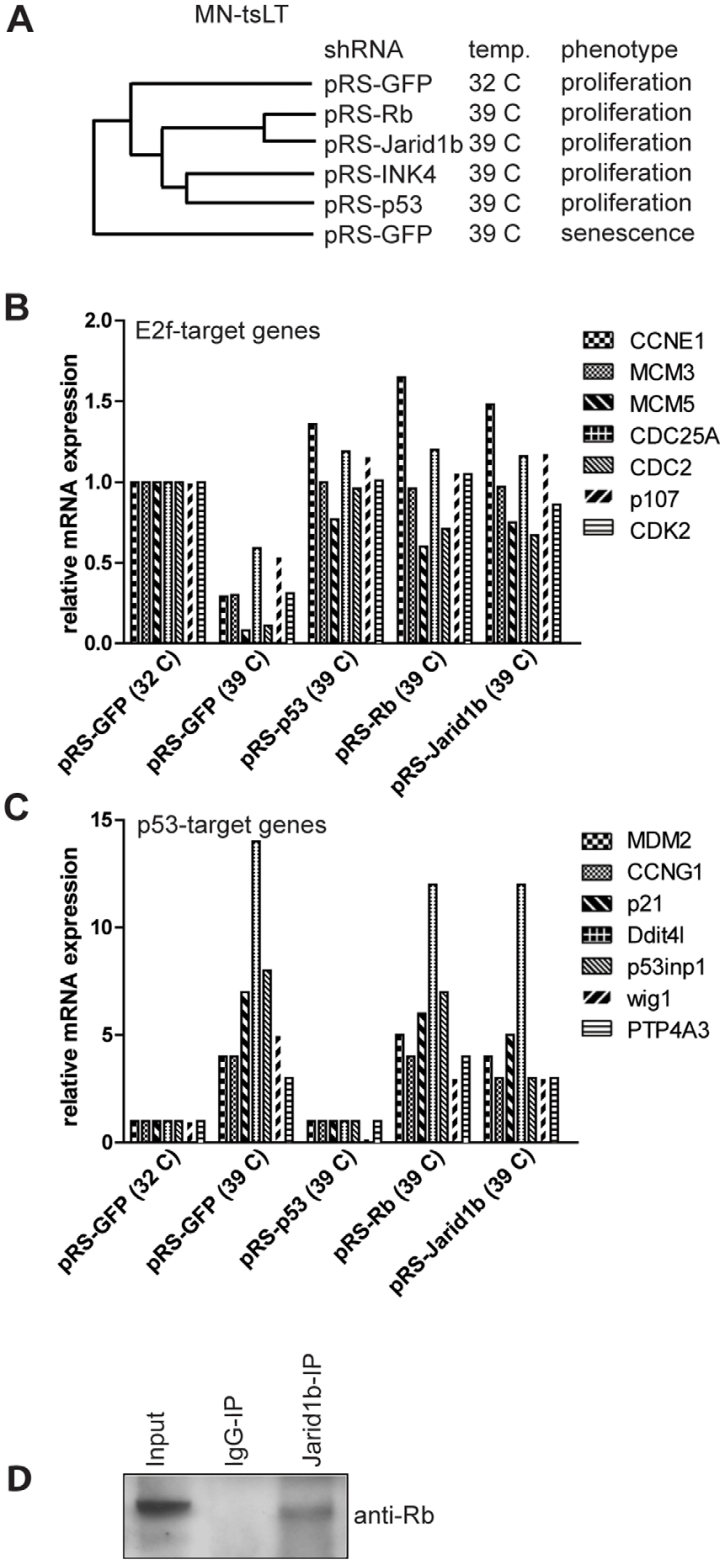
Jarid1b operates in the Rb pathway. (A) Unsupervised hierarchical clustering of mRNA expression of MN-tsLT cells stably transduced with the indicated knockdown vectors grown under the permissive or non-permissive temperature. (B) Relative expression of E2f-target genes from expression profiles from (A). (C) Relative expression of *p53* target genes from expression profiles from (A). Values were normalized to MN-tsLT cells cycling at 32°C. (D) Co-immunoprecipitation of Rb and Jarid1b. Protein fractions were isolated from MN-tsLT cells cultured for 4 days at 39°C followed by immunoprecipitation of Jarid1b. Immunoprecipitated samples were analyzed by western blot and probed with anti-Rb antibody.

Suppression of either the p16^INK4A^-Rb or the p19^ARF^-p53-p21^cip1^ pathways can mediate bypass of senescence in MN-tsLT cells (Figure 1A). To determine in which of these two pathways Jarid1b operates, we examined gene expression profiles of senescent MN-tsLT cells and MN-tsLT cells with knockdown of *p53*, *Rb1*, *Ink4a* (*Ink4a-Arf* locus) or the *Jarid1b* shRNA pool. Unsupervised hierarchical clustering of mRNA expression profiling revealed that the transcriptional profiles of *Jarid1b*-knockdown and *Rb*-knockdown cells were highly similar (Figure 3A), suggesting that Rb and Jarid1b may operate in the same pathway. Concordantly, expression of established E2f-target genes was downregulated in senescent cells but restored in *Rb1* and *Jarid1b*-knockdown cells similar to *p53*-knockdown cells (Figure 3B). To ask whether Jarid1b also functions in the p53 pathway we looked for the expression of *bona fide* p53-target genes in our micro-array data sets. As expected, p53-target genes were upregulated in senescent cells and downregulated in p53-knockdown cells (Figure 3C). In contrast, p53-target genes were induced in both *Rb1*-knockdown and *Jarid1b*-knockdown cells to a similar extent as in senescent MN-tsLT cells. These data may indicate that Jarid1b does not function in the p19^ARF^-p53-p21^cip1^ pathway. Moreover, *Jarid1b* is not a transcriptional target of p53 as knockdown of *p53* does not affect the expression of *Jarid1b* in MN-tsLT cells (Figure 2B).

Interestingly, it was previously reported that the protein product of a *JARID1B* splice variant binds to RB in co-immunoprecipitation experiments in MCF7 human breast cancer cells [34]. However, the functional significance of JARID1B in RB-mediated suppression of E2F-target genes was not explored. This is not a trivial question as over 150 proteins are known to interact with RB (www.hprd.org) but many of those do not modulate E2F-target gene expression. To further substantiate the interaction between Jarid1b and Rb, we performed a co-immunoprecipitation experiment in senescent MN-tsLT cells using an antibody against Jarid1b. Indeed we were able to detect endogenous Rb in the Jarid1b immunoprecipitation by western blotting using an Rb antibody, demonstrating that Jarid1b physically interacts with Rb in senescent MN-tsLT cells (Figure 3D). The expression data together with the interaction of Jarid1b and Rb may suggest that Jarid1b is involved in Rb-, but not p53, mediated execution of senescence in MN-tsLT cells.

### Jarid1b knockdown phenocopies loss of Rb in Rb-dependent senescence models

**Figure 4:**
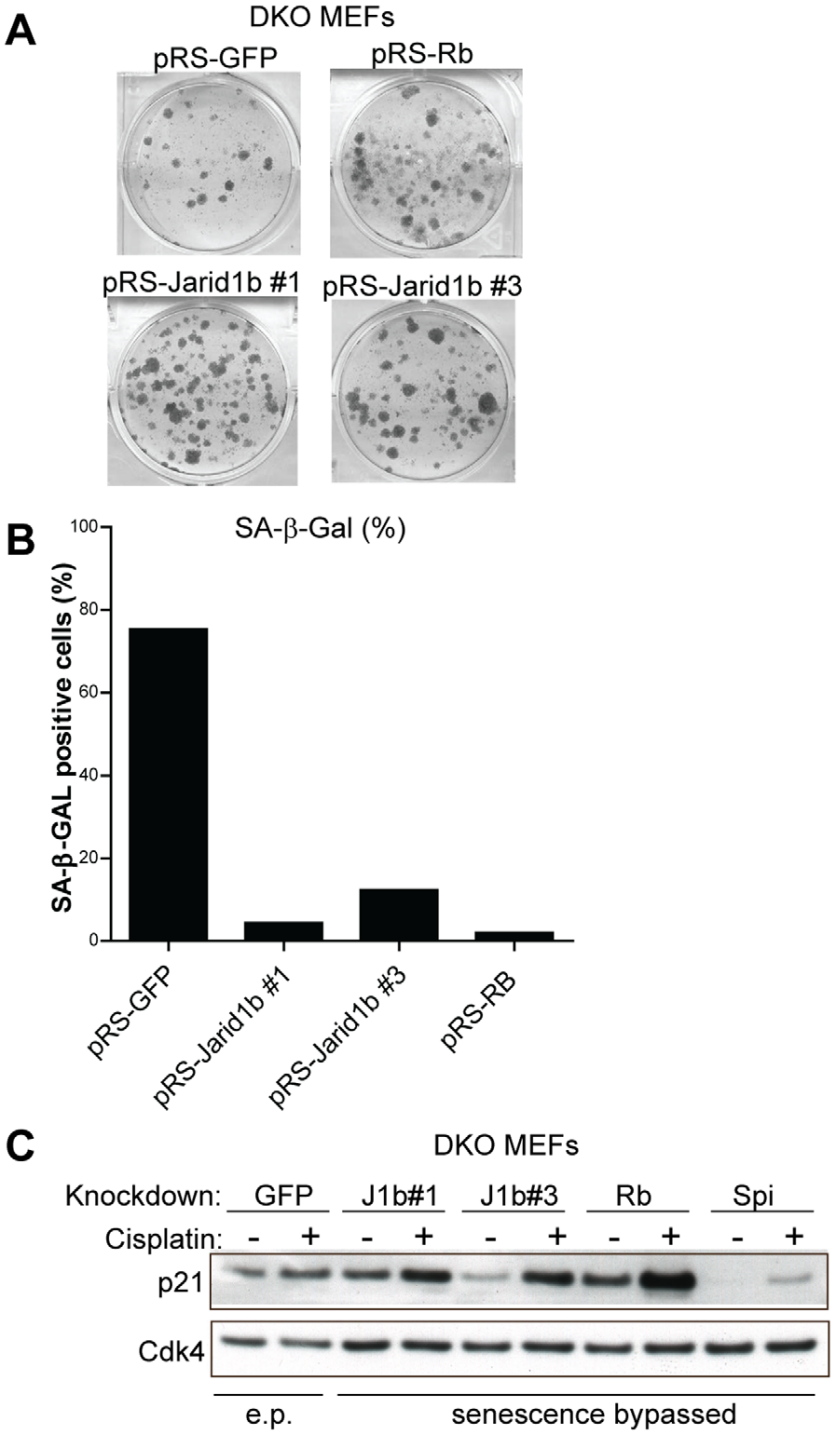
*Jarid1b*-knockdown is able to bypass cellular senescence in MEFs deficient for *Rbl1* and *Rbl2*. (A) MEFs, deficient for *Rbl1* and *Rbl2* (DKO MEFs), were transduced with functional *Jarid1b* or *Rb1* retroviral knockdown vectors and seeded for a colony formation assay. pRS-*GFP* was used as a negative control. (B) β-galactosidase quantification of DKO MEFs from (S2B). (C) Western blot analysis of p21^cip1^ in DKO MEFs from (A) treated with cisplatin o/n. pRS-*GFP* sample in first two lanes are early passage (e.p.) DKO MEFs. Spontaneously expanded colony outgrowths of pRS-*GFP* (SPi: spontaneously immortalized) transduced DKO MEFs from (A) were used as a control. Cdk4 staining serves as a loading control.

To confirm that Jarid1b functions in the Rb pathway we tested whether loss of *Jarid1b* could bypass senescence in another senescence model in which abrogation of the Rb pathway is sufficient for bypass. Primary MEFs with knockdown of *p53* are unable to undergo senescence whereas knockdown of *Rb1* does not result in bypass of senescence. Transduction of primary MEFs with the *Jarid1b* shRNA pool did not result in bypass of senescence (Supplementary Figure S2A). It has been shown previously that MEFs deficient for all three pocket proteins *Rb1*, *Rbl1* and *Rbl2* are unable to undergo senescence [35], [36]. In contrast, MEFs only deficient for *Rbl1* and *Rbl2* retain the ability to undergo senescence [37], suggesting that in these double knockout MEFs (DKO MEFs) Rb is the only retinoblastoma gene family member that executes the senescence program. We subsequently tested whether our knockdown vectors against *Jarid1b* could replace knockdown of *Rb1* to override cellular senescence in these DKO MEFs. Indeed, depletion of *Jarid1b* or *Rb1* prevented cellular senescence in DKO MEFs (Figure 4A). Unlike senescent DKO MEFs, *Rb1* and *Jarid1b*-knockdown cells did not stain positive for β-galactosidase (Figure 4B and Supplementary Figure S2B) and did not show a senescent morphology (Supplementary Figure S2C). Mutations in *Ink4a*, *Arf* and *p53* can lead to spontaneous immortalization of MEFs [1]. To exclude that *Jarid1b*-knockdown DKO MEFs were spontaneously immortalized (SPi), we assessed the status of the p53 pathway by treating cells with the DNA-damaging agent cisplatin and subsequently analyzed the expression of the p53 target gene *Cdkn1a* (Figure 4C). In contrast to SPi colonies derived from pRS-GFP transduced DKO MEFs, *Cdkn1a* expression was potently induced in *Jarid1b*-knockdown DKO MEFs after treatment with cisplatin. Collectively, these results show that *Jarid1b*-knockdown can phenocopy *Rb1*-knockdown in the bypass of cellular senescence in both MN-tsLT cells and DKO MEFs.

### Jarid1b associates with E2f-target genes during senescence

**Figure 5:**
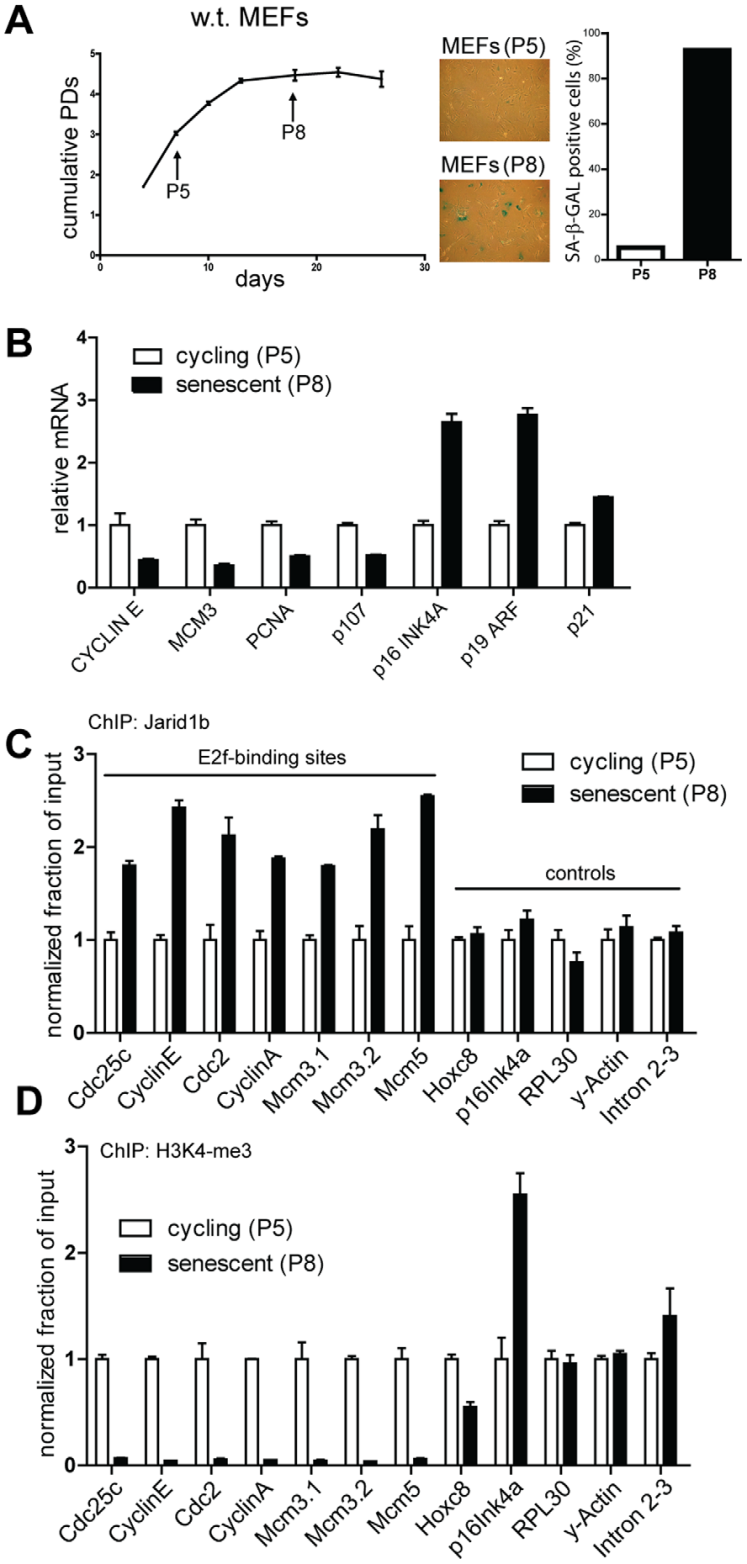
Jarid1b associates with the promoters of E2f-target genes during senescence. (A) Analysis of cycling, passage 5 (P5) and senescent, passage 8 (P8), primary MEFs for hallmarks of senescence. Shown are a proliferation curve of MEFs according to the 3T4 protocol (left panel, results shown as means ± SD), β–galactosidase staining of P5 and P8 MEFs (center panel) and quantification of β–galactosidase positive cells (right panel). (B) Analysis of mRNA expression of the indicated genes from P5 and P8 MEFs. Values are normalized to P5 and shown as means ± SD. (C) Jarid1b ChIP in P5 MEFs and P8 MEFs. The degree of enrichment at indicated promoters of E2f-target genes and control genes was measured by qPCR, non-specific binding of rabbit IgG controls was subtracted and results are presented as percentage of bound/input normalized to P5 samples for each gene. (D) H3K4me3 ChIP in P5 MEFs and P8 MEFs, performed as in (C). Non-specific binding of rabbit IgG controls was subtracted and quantification of H3K4me3 samples was normalized to H3-immunoprecipitations in the same experiment. PD: passage doubling, P: passage.

Using chromatin immunoprecipitation (ChIP) with an RB antibody followed by deep sequencing it was shown that RB associates with E2F-target genes involved in DNA replication and cell cycle progression in senescent diploid human fibroblasts [17]. RB orchestrates the senescence response by recruiting chromatin modifying enzymes to induce and maintain a repressive state of heterochromatin surrounding E2F-target genes [38], [39]. JARID1B has been shown to function as a transcriptional repressor by demethylating the active transcription mark H3K4me3 [40], [41]. We hypothesized that Jarid1b associates with Rb during senescence to remove the activating H3K4-trimethyl mark at promoters of E2f-target genes. To test whether Jarid1b associates with E2f-target genes during senescence we determined Jarid1b occupancy at E2f binding sites of E2f-target gene promoters in cycling and senescent MEFs by performing a ChIP experiment with an antibody specific for Jarid1b. We confirmed that MEFs at passage 8 (P8) were senescent as they displayed hallmarks of senescence that were not observed in passage 5 (P5) MEFs, such as positive staining for β-galactosidase, induction of senescence-associated tumor suppressor genes *Ink4a*, *Arf* and *Cdkn1a*, and downregulation of E2f-target genes *Ccne1*, *Mcm3*, *Pcna* and *Rbl1* (Figures 5A and B). In support of our hypothesis, we found an increased association of Jarid1b with promoters of E2f-target genes but not at promoters of control genes in senescent MEFs (Figure 5C). Next, we tested whether Jarid1b occupancy at E2f-target gene promoters was correlated with decreased H3K4 methylation at these promoters, by performing a ChIP with an antibody specific for H3K4me3 in the same chromatin factions. Indeed, we found that H3K4me3 was severely depleted at promoters of E2f-target genes in senescent cells (Figure 5D). Similar to MEFs, we observed an enhanced occupancy of Jarid1b at E2f-target gene promoters in senescent MN-tsLT cells associated with depletion of H3K4me3 levels (Supplementary Figure S3A and S3B). Taken together, these results demonstrate that there is increased binding of Jarid1b to E2f-target genes during senescence, which is correlated with a strong reduction of H3K4me3 of these E2f-target genes.

## Discussion

Chromatin is extensively modified during senescence to allow selective repression of E2F-target genes that control cellular proliferation. E2F-target gene promoters become targets for heterochromatin formation that are enriched for H3K9 methylation but depleted in H3K4 methylation [3], [13]. H3K4me3 is exclusively associated with the 5′ regions of practically all active genes whereas H3K9me3 is invariably enriched in transcriptionally silent regions [42], [43]. Several studies suggest that the formation of an epigenetic landscape that induces silencing of E2F-target genes during senescence is orchestrated by RB. In contrast to proteins responsible for H3K9 methylation of E2F-target genes, it is unknown which enzymes selectively demethylate H3K4me3 of E2F-target genes. Our data suggest that Jarid1b functions in a repressive complex with Rb to remove the H3K4 activation mark from E2f-target genes, a process that could contribute to their stable silencing during senescence in murine cells.

Recently, Lowe and colleagues, identified a non-redundant role for RB, but not p107 and p130, in promoting senescence by specifically repressing E2F-target genes involved in DNA replication [17], providing a rationale for why RB, but not its family members p107 and p130, is disabled in many, if not all, tumor cells [44]. Although near complete loss of RB may delay senescence induction [17], inactivation of Rb is not sufficient to bypass senescence in almost all models of senescence [4], [45]. We find here that suppression of *Jarid1b* can substitute for *Rb1* loss in override of senescence in mouse fibroblasts that can be bypassed by knockdown of *Rb1* alone, indicating a role for Jarid1b in the Rb pathway.

JARID1B has been implicated as an oncogene in breast and prostate cancer but as a tumor suppressor in melanoma, which may be attributed to tissue-specific regulation of genes that control carcinogenesis by JARID1B. For example, JARID1B was reported to transcriptionally regulate *BRCA1* in breast cancer, via direct interaction with promoter sites [40], [41], [46]. *JARID1B* is highly expressed in benign human melanocytic nevi, which invariably harbor oncogenic mutations but are protected from progressing into malignant tumors by oncogene-induced senescence [47], [48]. Importantly, it was found that the RB tumor suppressor network and not the p14^ARF^-p53-p21^cip1^ axis has a key role in the induction of senescence in naevi [48]. This study provided a rationale for the frequent genetic alterations in the p16^INK4A^-RB pathway in melanoma and the genetic predisposition of patients with germline mutations of the p16^INK4A^-RB tumor suppressor network to melanoma [49]. It was reported that RB recruits HDAC1, HP1β and SUV39H1 to induce senescence in naevi [39]. We speculate that JARID1B assists RB in senescent naevi to aid in the execution of senescence. Indeed, *JARID1B* is downregulated in malignant melanoma that progressed from a senescent naevus, while restoration of *JARID1B* expression in malignant melanoma inhibits proliferation [50]. It was recently found that in contrast to the bulk of melanoma tumor cells expressing very low levels of *JARID1B*, a small slow-growing subpopulation expresses high levels of *JARID1B*. The *JARID1B* expressing subpopulation was found to act as tumor-initiating cells, giving rise to highly proliferative progeny with low *JARID1B* expression [47]. We speculate that the high proliferation rate of melanoma cells with low *JARID1B* expression may be caused by depression of E2F-target genes and the consequential activation of the cell cycle.

In conclusion, we identified a novel component of the Rb-repressor complex that associates with E2f-target genes during senescence correlating with a strong decrease of H3K4me3 at the same promoters. Jarid1b binds to Rb in senescent cells and *Jarid1b*-knockdown can substitute for *Rb1*-knockdown in senescence models that are solely dependent on functional Rb. We speculate that one of the functions of Jarid1b is to repress E2f-target genes, providing a possible explanation for the differential expression of *JARID1B* in distinct tumors although additional research is needed to dissect the functional role of the plasticity in *JARID1B* expression in different tumor types.

## Materials and Methods

### shRNA Library Design

Design of oligonucleotides was done as previously described [51]. Multiple independent oligonucleotides (4 oligos/transcript) were designed to target the Jumonji C (JmjC)-domain-containing proteins, the lysine specific demethylase 1 (LSD1)-like family members, methyl CpG binding proteins and DNA methylases (see Supplemental table S1 for sequences). The oligonucleotides were pooled in 50 sets of 4 vectors, where each set of vectors was designed to target a single transcript, and cloned into the pRISC retroviral vector as previously described [51], [52]. More information and protocols on the oligo design and vector can be found at: http://www.screeninc.nl. (see Supplementary Table S1 for sequences).

### Cell Lines and Cell Culture

All cell lines were cultured in Dulbecco's modified Eagle's medium supplemented with 10% fetal calf serum and antibiotics. MN-tsLT mouse striatum cells express a mutant version of the huntingtin protein with an expanded polyglutamine repeat from a knock-in MN-tsLT allele and a stably introduced temperature sensitive mutant (tsA58) of SV40T antigen [19]. Primary MEFs deficient for the pocket proteins encoding genes *Rbl1* and *Rbl2* (DKO MEFs) were obtained from Dannenberg [37].

### Viral Transduction

Retroviral supernatants for each shRNA-pool were produced by transfection of phoenix packaging cells using the calcium-phosphate precipitation method. Forty-eight hours post-transfection, supernatants were harvested, filtered through a 0.45-µm filter and used for infection of target cells.

### shRNA library Screen, Colony Formation and Proliferation Assays

MN-tsLT cells grown at the permissive temperature (32°C) were seeded at a density of 2×10^5^ cells per well of a 6-well dish and used for viral infection the next day. After infection cells were selected with puromycin (1 µg/ml) for 2 days. The puromycin selected MN-tsLT cells were seeded at a density of 1×10^4^ cells per well of a 6-well dish and 6 hrs after plating shifted to the non-permissive temperature 39°C. After 2 weeks, cells were fixed with 4% formaldehyde, stained with 0.1% crystal violet and photographed. Crystal violet was extracted using 10% acetic acid and quantified at OD 590 nm.

Primary MEFs of FVB genetic background were transduced with retroviral shRNA constructs at passage 3, selected for two passages and subsequently seeded at a density of 1×10^4^ per 10 cm Ø dish for a colony formation assay. Cells were fixed and stained after 3 weeks. Growth curves were performed according to the 3T4 protocol and counted every 4 days. DKO MEFs (passage 2) were transduced with retroviral shRNA constructs at passage 3 and selected for two more passages with puromycin (1 µg/ml). Passage 5 DKO MEFs were seeded 1×10^4^ cells per well of a 6-well plate for a colony formation assay. Cells were fixed and stained after 2 weeks.

### Chromatin Immunoprecipitation

ChIP assays were performed using a commercially available ChIP assay kit (Simple ChIP Cell Signaling Technology, #9002) following the manufacturer's instructions. In short, MEFs and MN-tsLT cells were cultured in 15 cm Ø dishes and fixed with 1% formaldehyde (Sigma-Aldrich) for 10 min, followed by 2 washes with ice-cold PBS containing 1 mM PMSF. For each sample 4×10^7^ isolated nuclei were resuspended in 1 ml buffer B and treated with 6 µl micrococcal nuclease (2000 gel units/µl) for 20 min at 37°C, followed by sonication with a Branson Sonifier 250 for 3 times 10 s with 30 s-off interval times at output setting 2 for MEFs, and 5 times 15 s with 30 s-off interval times at output setting 2 for MN-tsLT cells. DNA was recovered from immune complexes on protein A-agarose beads with the following antibodies: Jarid1b (#3273, Cell Signaling Technology), H3K4me3 (ab1012, Abcam), H3 (#4620, Cell Signaling Technology) and normal rabbit IgG (#2729, Cell Signaling Technology). Real-time qPCR was performed using FastStart Universal SYBR Green Master (Roche) in a 7500 Fast Real-Time PCR System (Applied Biosystems). ChIP primers used are derived from Blais [53], Rowland [54] and Barradas [12] and listed in Supplementary Table S1. Data are presented as percentage of bound minus IgG controls divided by input and normalized to the proliferating condition (P5 for MEFs and 32°C for MN-tsLT cells) of 3 independent ChIPs on a single chromatin fraction. For MN-tsLT cells, the experiments were performed in 3 biological triplicates and for the MEFs in biological duplicates.

### Protein Extraction, Co-Immunoprecipitation and Western Blotting

Cells were lysed on ice with RIPA (150 mM NaCl, 50 mM Tris pH 8.0, 1% NP40, 0.5% sodium deoxycholate (DOC), 0.1% Sodium Dodecyl Sulfate (SDS)). Total protein (20–40 µg) was used for Western analysis with antibodies for p21 (F-5, Santa Cruz Biotechnology) and CDK4 (C-220, Santa Cruz Biotechnology). Co-immunoprecipitation was performed using total cell lysate of 8×10^6^ senescent MN-tsLT cells after culture for 4 days at 39°C. Binding of Jarid1b to Rb, was analyzed by immunoprecipitation with 8 µl Jarid1b (#3273, Cell Signaling Technology) in ELB (250 mM NaCl, 50 mM Hepes pH 7.3, 0.1% NP40 and Complete protease inhibitor cocktail from Roche) and subsequent Western analysis with an antibody against Rb (#554136, BD Pharmingen).

### Quantitative RT-qPCR

RNA was extracted using TriZOL (Invitrogen) according to the manufacture's protocol. cDNA synthesis and subsequent quantitative RT-PCR assays were performed to measure mRNA levels of genes using the 7500 Fast Real-Time PCR System as previously described [52]. Relative mRNA levels of each gene shown were normalized to the expression of *Rpl4*, *Gapdh*, *Actin* or *Rpl13* as indicated. The sequences for the primers for assays using SYBR Green master mix are listed in Supplementary Table S1.

### β-Galactosidase Staining

Cells were washed with PBS and fixed with 0.5% gluteraldehyde (in PBS) for 15′ at RT. Fixed cells were washed with PBS supplemented with 1 mM MgCL_2_. Cells were subsequently incubated 4–6 hrs (for MN-tsLT cells) or 10–12 hrs (for MEFs) at 37°C in staining solution (PBS pH 6.0, 5 mM K_4_Fe(CN)^6^ *3H_2_O, 5 mM K_3_Fe(CN)^6^, 1 mM MgCl_2_, 1 mg/ml X-Gal). All cells were processed simultaneously to allow comparison. A total of 1000 cells were counted per plate and scored for SA-β -Gal positive cells. For all SA-β -Gal stainings, the representative of at least two independent experiments is shown.

### Unsupervised Hierarchical Clustering

MN-tsLTcells were transduced with the indicated shRNAs, puromycin selected and shifted to the non-permissive temperature as indicated. RNA samples were made in TriZOL (Invitrogen) according to the manufacturer's instructions, RNA was cleaned with the RNeasy mini kit (#74106, Qiagen) and DNase treated with the RNase-free DNase Set (#79254, Qiagen) according to the manufacturer's protocols. RNA was amplified using the Illumina TotalPrep RNA amplification Kit (Part Number AMIL 1791) and subsequently hybridized to an Illumina HumanWG-6 V3 beadchip (BD-101-0603). Unsupervised hierarchical clustering analysis was performed after background subtraction and normalization with BeadStudio analysis software from Illumina.

## Supporting Information

Figure S1 *Jarid1b*-knockdown prevents senescence in MN-tsLT cells without affecting the induction of CDKNA1 expression. (A) Protein expression of Rb in senescent MN-tsLT cells transduced with a control vector (pRS-*GFP*) or an *Rb1*-knockdown vector (pRS-*Rb*). l.c.: loading control. (B) Brightfield images of MN-tsLT cells transduced with the indicated knockdown vectors and cultured at 39°C. pRS-*GFP* was used as a negative control. pRS-*p53*, and pRS-*Rb* were used as positive controls. The shRNA pool targetting *Jarid1b* and the two most potent *Jarid1b* knockdown vectors (#1 and #3) were tested. Cells transduced with negative control vectors show a typical round and flat morphology, characteristic of senescent cells. (C, D and E) RT-qPCR analysis shows relative mRNA expression of *Ccna1* (cyclin A) (C), *PcnA* (D) and *Cdkn1a* (E) as described in figure 2B.
(TIF)

Figure S2 *Jarid1b*-knockdown can replace *Rb1*-knockdown to prevent cellular senescence in *Rb1* ^wt^, *Rbl1* ^−/−^, *Rbl2* ^−/−^ (DKO) MEFs. (A) Colony formation assay of primary MEFs transduced with the indicated knockdown vectors. Late passage infected MEFs were seeded at low density in a 10 cm dish allowed for colony formation for 2 weeks and colonies were visualized by crystal violet. (B) β–galactosidase staining of DKO MEFs from Figure 4A. (C) Brightfield images of DKO MEFs transduced with the indicated constructs. As a negative control pRS-*GFP* was used. The negative control shows a round and flat morphology, which is typical of senescent cells.
(TIF)

Figure S3 Jarid1b associates with the promoters of E2f-target genes during senescence. (a) Jarid1b ChIP in MN-tsLT cells when cycling (32°C) or in senescence (39°C). The degree of enrichment at indicated promoters of E2f-target genes and control genes was measured by qPCR, non-specific binding of rabbit IgG controls was subtracted and results are presented as percentage of bound/input normalized to 32°C samples. (b) H3K4me3 ChIP in MN-tsLT cells when cycling (32°C) or in senescence (39°C), performed as in (a). Non-specific binding of rabbit IgG controls was subtracted and quantification of H3K4me3 samples was normalized to H3-immunoprecipitations performed in the same experiment on the same samples.
(TIF)

Table S1 Chromatin modifiers knockdown library. Sequences of knockdown vectors and sequences of primers are depicted.
(XLS)

## References

[1] Campisi J, d'Adda di Fagagna F (2007). Cellular senescence: when bad things happen to good cells.. Nat Rev Mol Cell Biol.

[2] Prieur A, Peeper DS (2008). Cellular senescence in vivo: a barrier to tumorigenesis.. Curr Opin Cell Biol.

[3] Adams PD (2007). Remodeling of chromatin structure in senescent cells and its potential impact on tumor suppression and aging.. Gene.

[4] Courtois-Cox S, Jones SL, Cichowski K (2008). Many roads lead to oncogene-induced senescence.. Oncogene.

[5] Macaluso M, Montanari M, Giordano A (2006). Rb family proteins as modulators of gene expression and new aspects regarding the interaction with chromatin remodeling enzymes.. Oncogene.

[6] Bracken AP, Kleine-Kohlbrecher D, Dietrich N, Pasini D, Gargiulo G (2007). The Polycomb group proteins bind throughout the INK4A-ARF locus and are disassociated in senescent cells.. Genes Dev.

[7] Dietrich N, Bracken AP, Trinh E, Schjerling CK, Koseki H (2007). Bypass of senescence by the polycomb group protein CBX8 through direct binding to the INK4A-ARF locus.. EMBO J.

[8] Pasini D, Hansen KH, Christensen J, Agger K, Cloos PA (2008). Coordinated regulation of transcriptional repression by the RBP2 H3K4 demethylase and Polycomb-Repressive Complex 2.. Genes Dev.

[9] Tzatsos A, Pfau R, Kampranis SC, Tsichlis PN (2009). Ndy1/KDM2B immortalizes mouse embryonic fibroblasts by repressing the Ink4a/Arf locus.. Proc Natl Acad Sci U S A.

[10] Pfau R, Tzatsos A, Kampranis SC, Serebrennikova OB, Bear SE (2008). Members of a family of JmjC domain-containing oncoproteins immortalize embryonic fibroblasts via a JmjC domain-dependent process.. Proc Natl Acad Sci U S A.

[11] Agger K, Cloos PA, Rudkjaer L, Williams K, Andersen G (2009). The H3K27me3 demethylase JMJD3 contributes to the activation of the INK4A-ARF locus in response to oncogene- and stress-induced senescence.. Genes Dev.

[12] Barradas M, Anderton E, Acosta JC, Li S, Banito A (2009). Histone demethylase JMJD3 contributes to epigenetic control of INK4a/ARF by oncogenic RAS.. Genes Dev.

[13] Narita M, Nunez S, Heard E, Lin AW, Hearn SA (2003). Rb-mediated heterochromatin formation and silencing of E2F target genes during cellular senescence.. Cell.

[14] Ferreira R, Naguibneva I, Pritchard LL, Ait-Si-Ali S, Harel-Bellan A (2001). The Rb/chromatin connection and epigenetic control: opinion.. Oncogene.

[15] Kuilman T, Michaloglou C, Mooi WJ, Peeper DS (2010). The essence of senescence.. Genes Dev.

[16] Braig M, Lee S, Loddenkemper C, Rudolph C, Peters AH (2005). Oncogene-induced senescence as an initial barrier in lymphoma development.. Nature.

[17] Chicas A, Wang X, Zhang C, McCurrach M, Zhao Z (2010). Dissecting the unique role of the retinoblastoma tumor suppressor during cellular senescence.. Cancer Cell.

[18] Talluri S, Isaac CE, Ahmad M, Henley SA, Francis SM (2010). A G1 checkpoint mediated by the retinoblastoma protein that is dispensable in terminal differentiation but essential for senescence.. Mol Cell Biol.

[19] Trettel F, Rigamonti D, Hilditch-Maguire P, Wheeler VC, Sharp AH (2000). Dominant phenotypes produced by the HD mutation in STHdh(Q111) striatal cells.. Hum Mol Genet.

[20] Brummelkamp TR, Kortlever RM, Lingbeek M, Trettel F, MacDonald ME (2002). TBX-3, the gene mutated in Ulnar-Mammary Syndrome, is a negative regulator of p19ARF and inhibits senescence.. J Biol Chem.

[21] Dirac AM, Bernards R (2003). Reversal of senescence in mouse fibroblasts through lentiviral suppression of p53.. J Biol Chem.

[22] Epping MT, Meijer LA, Krijgsman O, Bos JL, Pandolfi PP (2011). TSPYL5 suppresses p53 levels and function by physical interaction with USP7.. Nat Cell Biol.

[23] Shi Y, Whetstine JR (2007). Dynamic regulation of histone lysine methylation by demethylases.. Mol Cell.

[24] Kouzarides T (2007). Chromatin modifications and their function.. Cell.

[25] Vogt T, Kroiss M, McClelland M, Gruss C, Becker B (1999). Deficiency of a novel retinoblastoma binding protein 2-homolog is a consistent feature of sporadic human melanoma skin cancer.. Lab Invest.

[26] Lu PJ, Sundquist K, Baeckstrom D, Poulsom R, Hanby A (1999). A novel gene (PLU-1) containing highly conserved putative DNA/chromatin binding motifs is specifically up-regulated in breast cancer.. J Biol Chem.

[27] Christensen J, Agger K, Cloos PA, Pasini D, Rose S (2007). RBP2 belongs to a family of demethylases, specific for tri-and dimethylated lysine 4 on histone 3.. Cell.

[28] Iwase S, Lan F, Bayliss P, de la Torre-Ubieta L, Huarte M (2007). The X-linked mental retardation gene SMCX/JARID1C defines a family of histone H3 lysine 4 demethylases.. Cell.

[29] Klose RJ, Yan Q, Tothova Z, Yamane K, Erdjument-Bromage H (2007). The retinoblastoma binding protein RBP2 is an H3K4 demethylase.. Cell.

[30] Seward DJ, Cubberley G, Kim S, Schonewald M, Zhang L (2007). Demethylation of trimethylated histone H3 Lys4 in vivo by JARID1 JmjC proteins.. Nat Struct Mol Biol.

[31] Xiang Y, Zhu Z, Han G, Ye X, Xu B (2007). JARID1B is a histone H3 lysine 4 demethylase up-regulated in prostate cancer.. Proc Natl Acad Sci U S A.

[32] Cloos PA, Christensen J, Agger K, Helin K (2008). Erasing the methyl mark: histone demethylases at the center of cellular differentiation and disease.. Genes Dev.

[33] Echeverri CJ, Beachy PA, Baum B, Boutros M, Buchholz F (2006). Minimizing the risk of reporting false positives in large-scale RNAi screens.. Nat Methods.

[34] Roesch A, Becker B, Meyer S, Wild P, Hafner C (2005). Retinoblastoma-binding protein 2-homolog 1: a retinoblastoma-binding protein downregulated in malignant melanomas.. Mod Pathol.

[35] Dannenberg JH, van Rossum A, Schuijff L, te Riele H (2000). Ablation of the retinoblastoma gene family deregulates G(1) control causing immortalization and increased cell turnover under growth-restricting conditions.. Genes Dev.

[36] Sage J, Mulligan GJ, Attardi LD, Miller A, Chen S (2000). Targeted disruption of the three Rb-related genes leads to loss of G(1) control and immortalization.. Genes Dev.

[37] Dannenberg JH, Schuijff L, Dekker M, van der Valk M, te Riele H (2004). Tissue-specific tumor suppressor activity of retinoblastoma gene homologs p107 and p130.. Genes Dev.

[38] Narita M, Krizhanovsky V, Nunez S, Chicas A, Hearn SA (2006). A novel role for high-mobility group a proteins in cellular senescence and heterochromatin formation.. Cell.

[39] Bandyopadhyay D, Curry JL, Lin Q, Richards HW, Chen D (2007). Dynamic assembly of chromatin complexes during cellular senescence: implications for the growth arrest of human melanocytic nevi.. Aging Cell.

[40] Scibetta AG, Santangelo S, Coleman J, Hall D, Chaplin T (2007). Functional analysis of the transcription repressor PLU-1/JARID1B.. Mol Cell Biol.

[41] Yamane K, Tateishi K, Klose RJ, Fang J, Fabrizio LA (2007). PLU-1 is an H3K4 demethylase involved in transcriptional repression and breast cancer cell proliferation.. Mol Cell.

[42] Ruthenburg AJ, Allis CD, Wysocka J (2007). Methylation of lysine 4 on histone H3: intricacy of writing and reading a single epigenetic mark.. Mol Cell.

[43] Mikkelsen TS, Ku M, Jaffe DB, Issac B, Lieberman E (2007). Genome-wide maps of chromatin state in pluripotent and lineage-committed cells.. Nature.

[44] Burkhart DL, Sage J (2008). Cellular mechanisms of tumour suppression by the retinoblastoma gene.. Nat Rev Cancer.

[45] Serrano M, Lin AW, McCurrach ME, Beach D, Lowe SW (1997). Oncogenic ras provokes premature cell senescence associated with accumulation of p53 and p16INK4a.. Cell.

[46] Tan K, Shaw AL, Madsen B, Jensen K, Taylor-Papadimitriou J (2003). Human PLU-1 Has transcriptional repression properties and interacts with the developmental transcription factors BF-1 and PAX9.. J Biol Chem.

[47] Roesch A, Fukunaga-Kalabis M, Schmidt EC, Zabierowski SE, Brafford PA (2010). A temporarily distinct subpopulation of slow-cycling melanoma cells is required for continuous tumor growth.. Cell.

[48] Michaloglou C, Vredeveld LC, Soengas MS, Denoyelle C, Kuilman T (2005). BRAFE600-associated senescence-like cell cycle arrest of human naevi.. Nature.

[49] Halaban R (2005). Rb/E2F: a two-edged sword in the melanocytic system.. Cancer Metastasis Rev.

[50] Roesch A, Becker B, Schneider-Brachert W, Hagen I, Landthaler M (2006). Re-expression of the retinoblastoma-binding protein 2-homolog 1 reveals tumor-suppressive functions in highly metastatic melanoma cells.. J Invest Dermatol.

[51] Berns K, Hijmans EM, Mullenders J, Brummelkamp TR, Velds A (2004). A large-scale RNAi screen in human cells identifies new components of the p53 pathway.. Nature.

[52] Huang S, Laoukili J, Epping MT, Koster J, Holzel M (2009). ZNF423 is critically required for retinoic acid-induced differentiation and is a marker of neuroblastoma outcome.. Cancer Cell.

[53] Blais A, van Oevelen CJ, Margueron R, Acosta-Alvear D, Dynlacht BD (2007). Retinoblastoma tumor suppressor protein-dependent methylation of histone H3 lysine 27 is associated with irreversible cell cycle exit.. J Cell Biol.

[54] Rowland BD, Denissov SG, Douma S, Stunnenberg HG, Bernards R (2002). E2F transcriptional repressor complexes are critical downstream targets of p19(ARF)/p53-induced proliferative arrest.. Cancer Cell.

