# Supplementary figures and images for: The Histone Demethylase Jarid1b (Kdm5b) Is a Novel Component of the Rb Pathway and Associates with E2f-Target Genes in MEFs during Senescence
Source: PLoS One. 2011 Sep 27;6(9):e25235. doi: 10.1371/journal.pone.0025235 (PMC3181323; doi:10.1371/journal.pone.0025235)

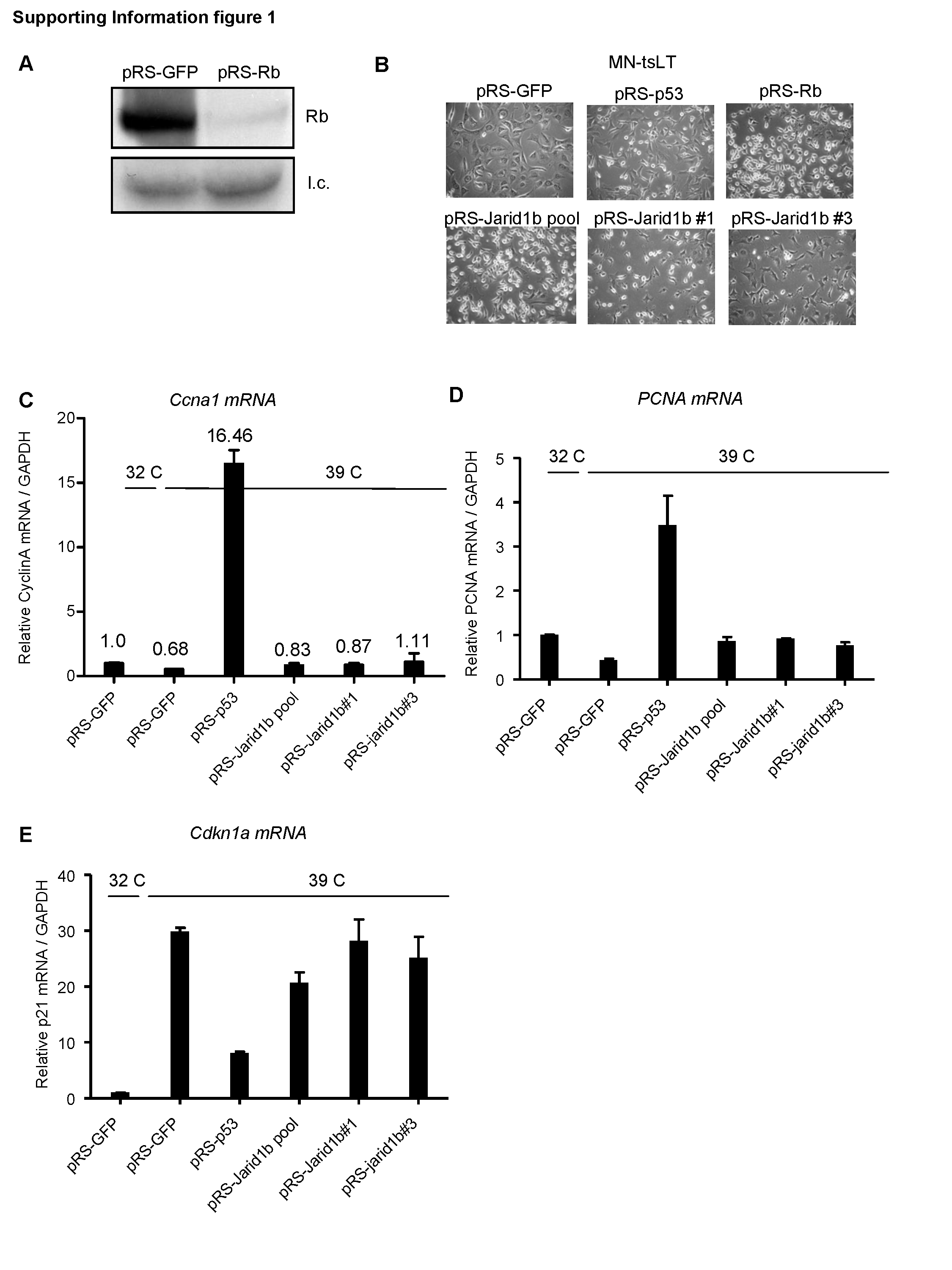

Supplement: Figure S1 — Jarid1b-knockdown prevents senescence in MN-tsLT cells without affecting the induction of CDKNA1 expression. (A) Protein expression of Rb in senescent MN-tsLT cells transduced with a control vector (pRS-GFP) or an Rb1-knockdown vector (pRS-Rb). l.c.: loading control. (B) Brightfield images of MN-tsLT cells transduced with the indicated knockdown vectors and cultured at 39°C. pRS-GFP was used as a negative control. pRS-p53, and pRS-Rb were used as positive controls. The shRNA pool targetting Jarid1b and the two most potent Jarid1b knockdown vectors (#1 and #3) were tested. Cells transduced with negative control vectors show a typical round and flat morphology, characteristic of senescent cells. (C, D and E) RT-qPCR analysis shows relative mRNA expression of Ccna1 (cyclin A) (C), PcnA (D) and Cdkn1a (E) as described in figure 2B. (TIF) [file pone.0025235.s001.tif]

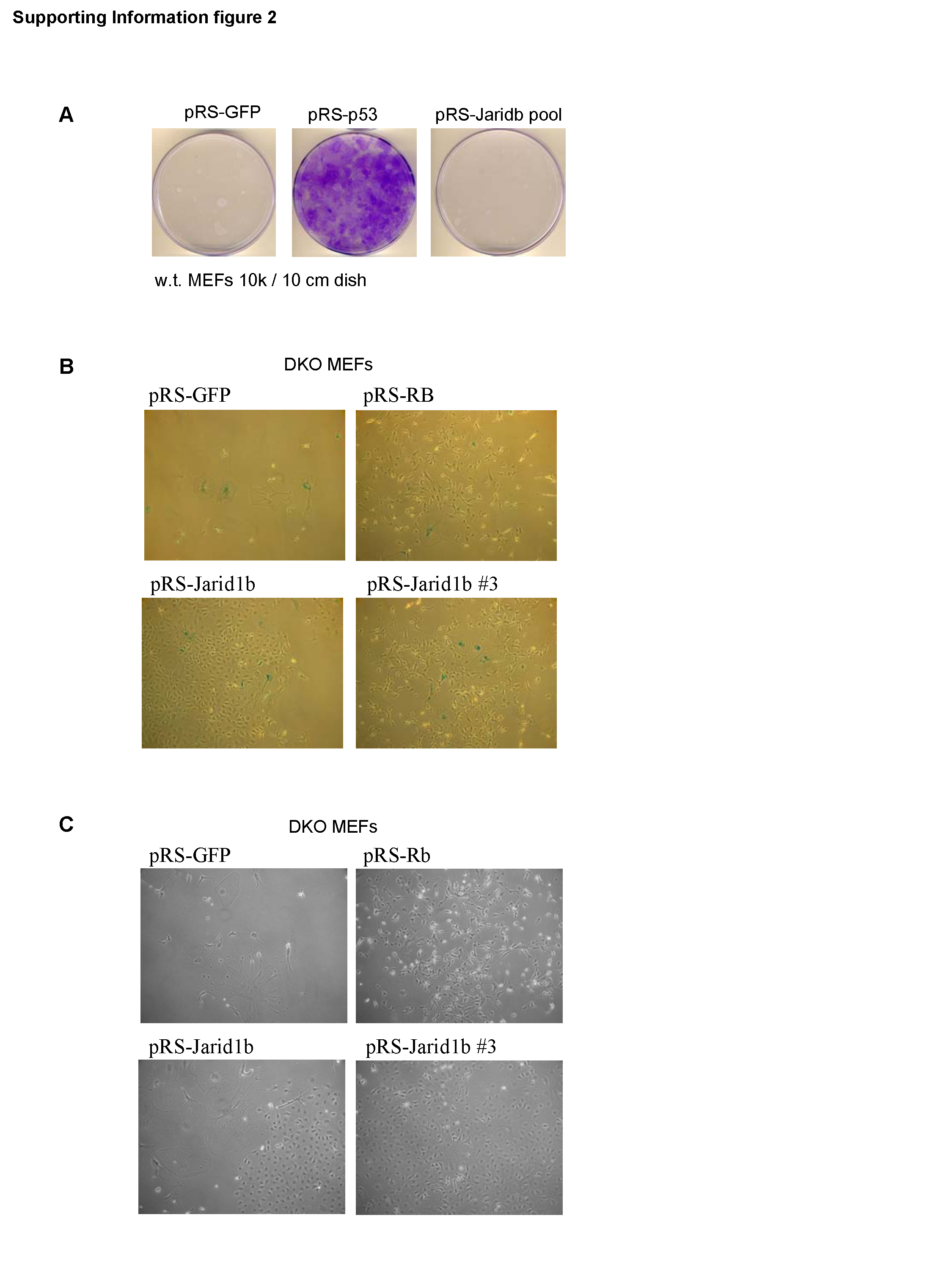

Supplement: Figure S2 — Jarid1b-knockdown can replace Rb1-knockdown to prevent cellular senescence in Rb1 wt, Rbl1 −/−, Rbl2 −/− (DKO) MEFs. (A) Colony formation assay of primary MEFs transduced with the indicated knockdown vectors. Late passage infected MEFs were seeded at low density in a 10 cm dish allowed for colony formation for 2 weeks and colonies were visualized by crystal violet. (B) β–galactosidase staining of DKO MEFs from Figure 4A. (C) Brightfield images of DKO MEFs transduced with the indicated constructs. As a negative control pRS-GFP was used. The negative control shows a round and flat morphology, which is typical of senescent cells. (TIF) [file pone.0025235.s002.tif]

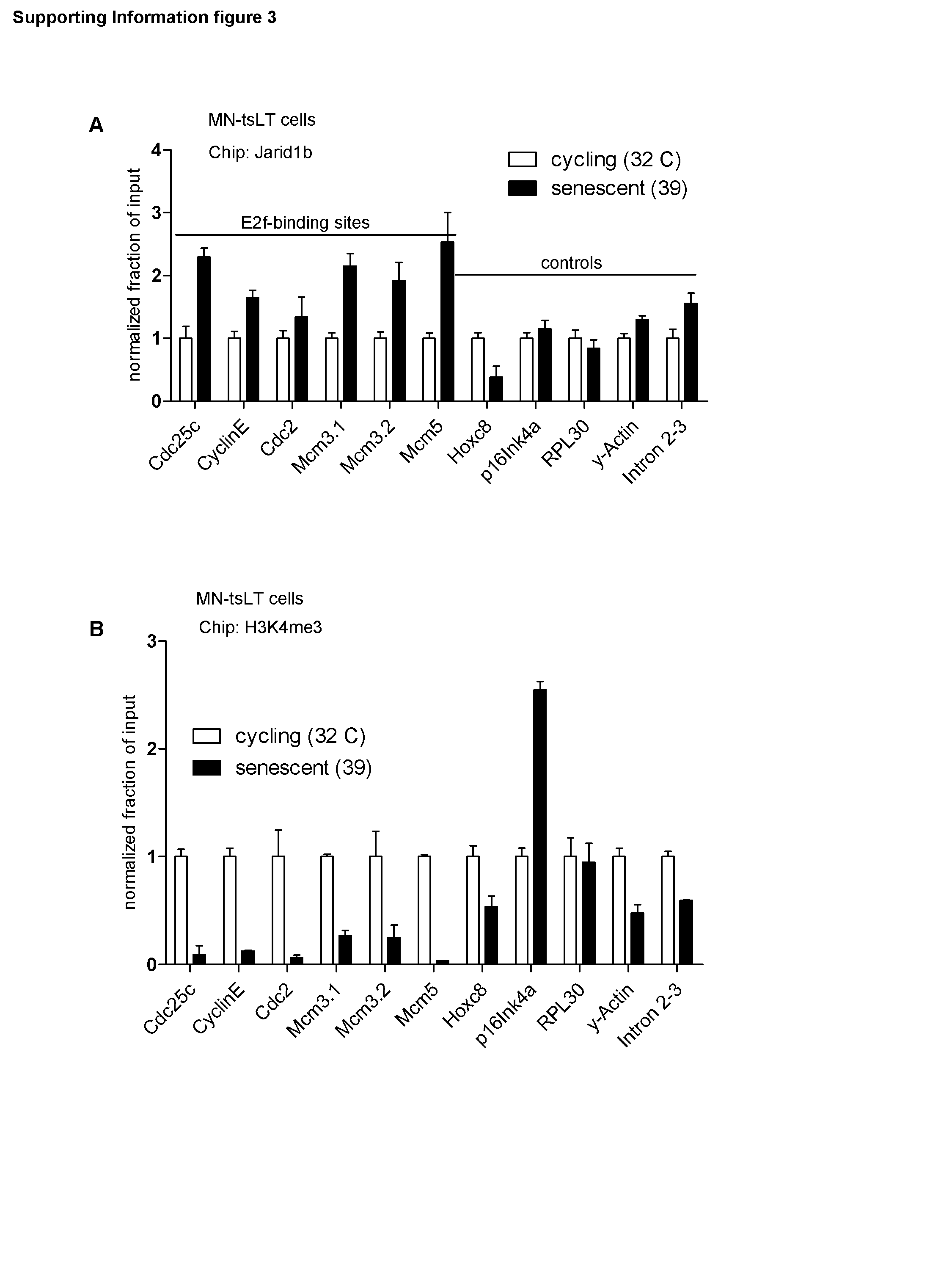

Supplement: Figure S3 — Jarid1b associates with the promoters of E2f-target genes during senescence. (a) Jarid1b ChIP in MN-tsLT cells when cycling (32°C) or in senescence (39°C). The degree of enrichment at indicated promoters of E2f-target genes and control genes was measured by qPCR, non-specific binding of rabbit IgG controls was subtracted and results are presented as percentage of bound/input normalized to 32°C samples. (b) H3K4me3 ChIP in MN-tsLT cells when cycling (32°C) or in senescence (39°C), performed as in (a). Non-specific binding of rabbit IgG controls was subtracted and quantification of H3K4me3 samples was normalized to H3-immunoprecipitations performed in the same experiment on the same samples. (TIF) [file pone.0025235.s003.tif]
